# Prediction of estimated glomerular filtration rate slope and kidney prognosis of patients with chronic kidney disease

**DOI:** 10.1038/s41598-026-38246-8

**Published:** 2026-02-17

**Authors:** Hajime Nagasu, Takaya Nakashima, Katsuhito Ihara, Ryo Fujimori, Tadahiro Goto, Daisuke Nitta, Seiji Kishi, Tamaki Sasaki, Naoki Kashihara

**Affiliations:** 1 Department of Nephrology and Hypertension, Kawasaki Medical School, 577 Matsushima, Kurashiki, 701-0192 Okayama Japan; 2 Department of Anesthesiology and Intensive Care Medicine, Nagasaki University Graduate School of Biomedical Sciences, Nagasaki, Japan; 3 TXP Medical Co. Ltd, Tokyo, Japan; 4 Medicine Division, Nippon Boehringer Ingelheim Co. Ltd, Tokyo, Japan; 5 Department of Health Data Science, Graduate School of Data Science, Yokohama City University, Yokohama, Japan; 6 Kawasaki Geriatric Medical Center, Kawasaki Medical School, Okayama, Japan

**Keywords:** Diseases, Medical research, Nephrology

## Abstract

Chronic kidney disease (CKD) is a significant global health challenge, yet the application of eGFR slope as a metric for CKD progression remains underdeveloped in primary care settings. Using data from J-CKD-DB-Ex, Japan’s largest CKD database, we developed and validated a machine learning-based model to predict eGFR slope. The study included 10,474 patients aged ≥ 18 years with eGFR < 60 mL/min/1.73 m² or proteinuria at baseline. The median age of participants was 69.0 years [IQR: 62.0–77.0], and 52% (5,493/10,474) of the cohort were male. The Median baseline eGFR was 52.7 mL/min/1.73 m² [IQR: 44.7–57.8]. Predictors included demographic, clinical, and laboratory data. We compared three models: linear regression, LightGBM, and LSTM networks. Among 10,474 patients (median age 69.0 years), the LightGBM model achieved superior performance (RMSE = 2.95 mL/min/1.73 m²/year) compared to LSTM (RMSE = 3.94) and conventional linear regression (RMSE = 15.87). The model was implemented as a web-based application for clinical use. This machine learning-based prediction model achieves superior accuracy in estimating eGFR trajectory and enables real-time prediction using single time-point data. The web-based tool supports early identification of high-risk patients, enabling timely interventions and specialist referrals in primary care settings.

**Supplementary Information:**

The online version contains supplementary material available at 10.1038/s41598-026-38246-8.

## Introduction

Since the concept of chronic kidney disease (CKD) was established in 2002, the importance of early diagnosis and treatment for kidney dysfunction has been widely recognized. However, the global number of patients requiring kidney replacement therapy continues to rise. Furthermore, CKD is a significant risk factor for cardiovascular disease (CVD) and mortality, posing a major public health challenge^1,2^. An epidemiological survey conducted in 2005 estimated that 12.9% of Japanese adults, or approximately 13.3 million people, had CKD. However, when considering the higher prevalence of CKD among individuals who do not undergo health checkups compared to those who do, a revised estimate suggests that one in five Japanese adults, or approximately 20 million people, may have CKD^3^. Given the substantial number of patients with CKD, it is impractical to manage all cases exclusively with nephrologists. As a result, the majority of individuals with early-stage CKD are managed mainly by primary care physicians (PCPs), whereas those with CKD stage G3b or more advanced disease are typically referred to nephrologists for specialized care. However, awareness of CKD and its early detection remains insufficient, as does the establishment of collaborative medical systems between PCPs and nephrologists in various regions.

For advanced CKD (G3b–G5 in CKD staging), a 30–40% decline in eGFR or a doubling of serum creatinine levels was established as a surrogate endpoint for end-stage kidney disease (ESKD) by Kidney Disease: Improving Global Outcomes (KDIGO) in 2017^4^. These surrogate endpoints were validated in Japanese populations and incorporated into clinical guidelines for CKD evaluation^5^. In 2018, a scientific workshop sponsored by the National Kidney Foundation, U.S. Food and Drug Administration, and European Medicines Agency recommended the use of a surrogate endpoint for early CKD^6^. These findings highlight the value of assessing eGFR slope in CKD management.

However, the integration of this approach into clinical practice by PCPs remains limited. Empowering PCPs to accurately predict CKD progression using routine clinical data is essential for advancing CKD prevention, facilitating early detection, and optimizing the timing of referrals to specialists.

At present, several representative CKD prognosis prediction models have been developed. While these models enable risk assessment, none are based on the eGFR slope. Monitoring changes in eGFR trajectory and utilizing the eGFR slope could provide PCPs with a valuable tool for interpreting kidney function trends and integrating these insights into routine clinical practice.

Notable CKD prognosis prediction models primarily focus on assessing the risk of progression to ESKD. While these models provide valuable insights for long-term risk stratification, tools capable of precisely forecasting future eGFR levels remain underdeveloped. This gap is significant, as predicting kidney function trajectory with precision would allow PCPs to anticipate CKD progression better and optimize patient management. Tracking changes in eGFR trajectory, mainly through the eGFR slope, could bridge this gap. The eGFR slope offers a dynamic and actionable metric that not only aids in interpreting kidney function prognosis but also supports PCPs in incorporating these insights into their daily clinical practice. This approach has the potential to improve early detection　and facilitate more effective collaboration with specialists by offering a more precise projection of future kidney function.

Using data from the J-CKD-DB-Ex, the largest longitudinal CKD database in Japan, we aimed to develop and validate a prognosis prediction model based on the eGFR slope. The J-CKD-DB-Ex is a real-world database tailored explicitly to CKD in Japan, offering comprehensive and nationally representative data. By systematically collecting information from hospitals nationwide, without significant regional biases, this database provides a detailed overview of CKD management practices in Japan. Its ability to capture the state of CKD diagnosis and treatment across diverse clinical settings makes it an invaluable resource for research. Utilizing this robust and representative dataset enhances the relevance and applicability of our prediction model to real-world clinical scenarios in Japan. The goal of this study is to estimate the extent of future kidney function decline by projecting the eGFR slope from current clinical information and predicting the trajectory 2–3 years into the future. Unlike conventional kidney failure prediction models, this eGFR slope prediction model is designed specifically for the context of daily clinical practice among PCPs. Additionally, it allows for on-demand predictions at any given point based on historical health data. By supporting disease awareness and fostering interdisciplinary collaboration, this model is expected to facilitate the prevention and early detection of CKD in primary care settings. This gap is significant, as accurate prediction of kidney function trajectories would allow PCPs to better anticipate CKD progression and optimise patient management. However, a major barrier to incorporating eGFR slope into routine clinical practice is the requirement for multiple eGFR measurements over time. In primary care settings, longitudinal data are often lacking or collected inconsistently. Therefore, the ability to predict eGFR slope from a single time-point measurement - combined with other routinely available clinical parameters - represents a significant advance. This feature greatly enhances the practicality and applicability of the model in real-world clinical settings, particularly for PCPs managing early-stage CKD. The eGFR slope provides a dynamic and actionable metric that not only aids in the interpretation of renal function prognosis, but also facilitates earlier detection and more effective collaboration between PCPs and specialists.

## Methods

### Study design and ethics

This is a non-interventional, retrospective, prognostic study to develop and validate machine learning models for predicting eGFR slope, using a large, multicenter registry of CKD. This study was approved by the ethics committee of Kawasaki Medical School. Owing to the nature of this study, it was exempt from the need to obtain informed consent from participants (Nos. 6400) and in accordance with the principles of the Declaration of Helsinki. Because of the deidentified nature of patient records, informed consent was obtained through an opt-out method on the web site of each participating university hospital in accordance with the Ethical Guidelines for Medical and Health Research Involving Human Subjects in Japan.

### Data source

J-CKD-DB-Ex is the largest, longitudinal (for seven years) database of CKD in Japan.

**Fig. 1:**
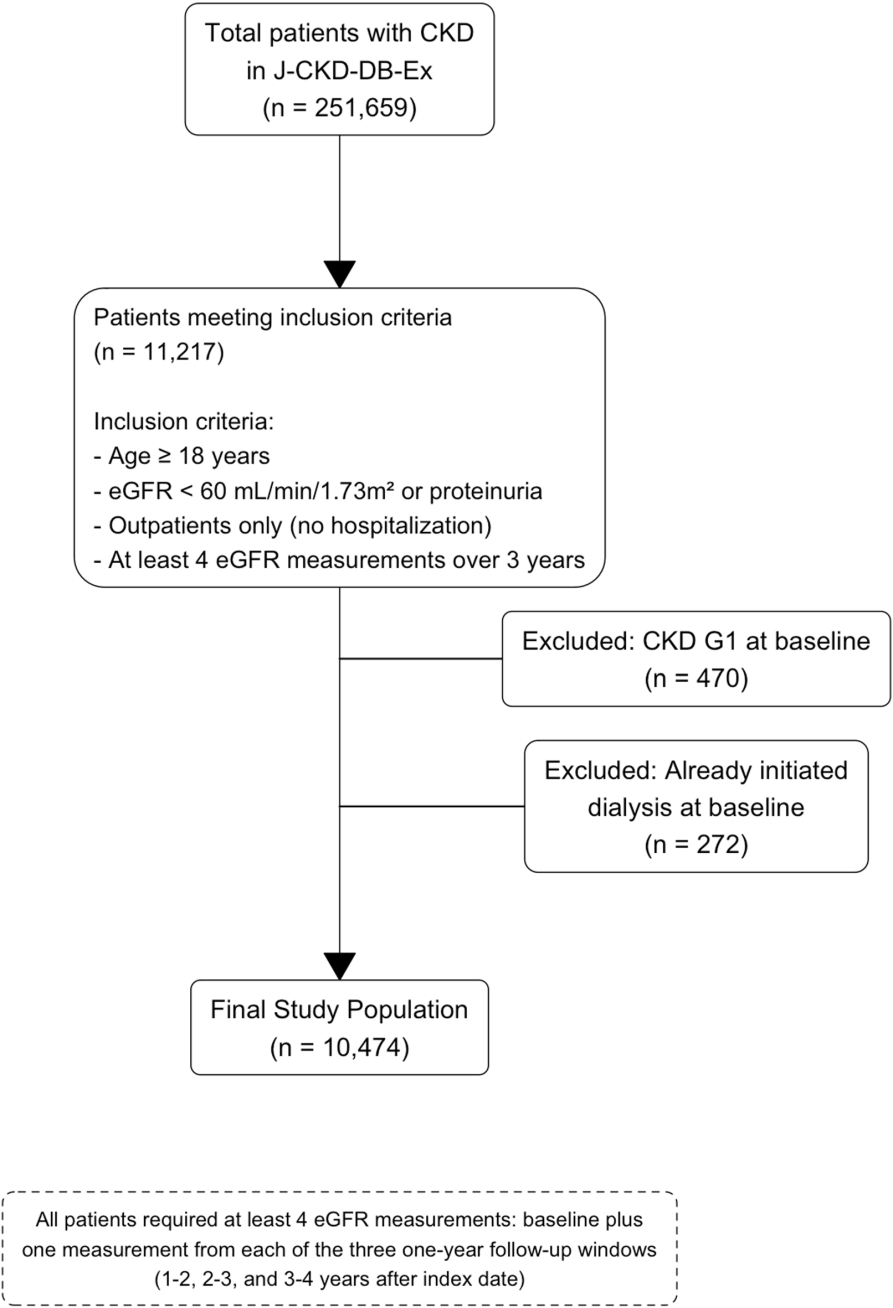
Patients flow of this study. The patient data flow used in this study is show. The study used data from J-CKD-DB-Ex from 2014 to 2022. eGFR, estimated glomerular filtration rate.

The Ministry of Health, Labor and Welfare has developed the Standardized Structured Medical Information eXchange (SS-MIX2) system, which streamlines the compilation of Electronic Health Record (EHR) data from various systems. The Japanese Society of Nephrology and the Japan Association for Medical Informatics have established a comprehensive clinical database for patients with CKD, known as the Japan Chronic Kidney Disease Database (J-CKD-DB-Ex), by leveraging the SS-MIX2 system^7,8^. J-CKD-DB-Ex included patients with CKD aged 18 years or older who had either proteinuria ≥ 1 by dipstick test or eGFR < 60 mL/min/1.73 m^2^. Currently, the database is being further expanded with the participation of 15 university hospitals and has registered approximately 250,000 cases of CKD to date. Patients flow was shown in Fig. 1.

### Study population

**Fig. 2:**
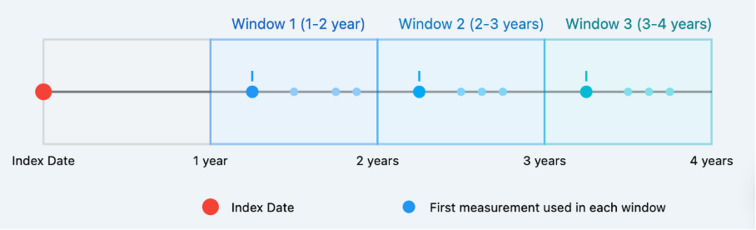
Definition of measurement windows and data collection points for eGFR. Study timeline depicting the index date and follow-up measurement windows for eGFR data collection. Abbreviations: eGFR, estimated Glomerular Filtration Rate.

We used J-CKD-DB-Ex, which is CKD patients database. J-CKD-DB-Ex included patients with CKD aged 18 years or older who had either proteinuria ≥ 1 by dipstick test or eGFR < 60 mL/min/1.73 m^2^. I extracted and used only the necessary patient data from this database. This study included only outpatients who had never been hospitalized throughout the study period, as it was designed to predict outcomes in an outpatient setting. For eGFR measurements, we set one-year windows starting from the initial eGFR measurement (index date) and adopted the first measurement within each window. Specifically, patients were required to have at least one eGFR measurement during each of three one-year windows: 1–2 years, 2–3 years, and 3–4 years after the index date. Therefore, during the observation period, a minimum of four measurements was required, consisting of the index date measurement plus one measurement from each of these three windows (Fig. 2).

We excluded patients with (1) CKD G1 at baseline, (2) who had already initiated dialysis at baseline.

### Predictors

We used the following baseline predictors: gender (binary), age (continuous, years), baseline eGFR (continuous, mL/min/1.73 m²), serum creatinine (continuous, mg/dL), serum albumin (continuous, g/dL), serum sodium (continuous, mEq/L), serum potassium (continuous, mEq/L), qualitative urine protein by dipstick test (binary, positive/negative), quantitative urine protein (continuous, g/gCr), diabetes mellitus and hypertension diagnoses defined by International Classification of Diseases 10th revision (ICD-10) codes (both binary), and medication use including renin-angiotensin system (RAS) inhibitors, sodium-glucose cotransporter-2 (SGLT2) inhibitors, and mineralocorticoid receptor (MR) blockers (all binary). All predictors are used for LightGBM and LSTM.

### Outcomes

The primary outcome is the eGFR slope, defined as the difference between the baseline eGFR and follow-up eGFR values over time, expressed in mL/min/1.73 m²/year. To calculate the eGFR slope, we used eGFR measurements obtained at 1, 2, and 3 years after baseline. The eGFR values were calculated using the Japanese GFR estimation equation based on serum creatinine values^9^.

### Prediction methods and statistical analysis

We developed and validated prediction models as follows:

1. Data splitting.
2. Definition of baseline data and outcomes.
3. Model development in the training set.
4. Hyperparameter tuning.
5. Model evaluation.

### Data splitting

We first randomly split the entire dataset into training (80%) and test (20%) sets. The test set was held out and used only for the final evaluation of the model’s performance. For model development and validation, we employed 5-fold cross-validation on the training set, where the training data was divided into five equal parts. In each fold of the cross-validation, one part served as a validation set for hyperparameter tuning and model selection, while the remaining four parts were used for training. This process was repeated five times, with each fold serving as the validation set once, ensuring that every sample in the training data contributed to both model training and validation. The test data was completely isolated and not used in any part of model training or tuning, but reserved solely for the final performance evaluation.

### Definition of baseline data and outcomes

・Predictors and missing data handling.

We used the following baseline predictors: gender (binary), age (continuous, years), baseline eGFR (continuous, mL/min/1.73 m²), serum creatinine (continuous, mg/dL), serum albumin (continuous, g/dL), serum sodium (continuous, mEq/L), serum potassium (continuous, mEq/L), qualitative urine protein by dipstick test (binary, positive/negative), quantitative urine protein (continuous, g/gCr), diabetes mellitus and hypertension diagnoses defined by International Classification of Diseases 10th revision (ICD-10) codes (both binary), and medication use including renin-angiotensin system (RAS) inhibitors, sodium-glucose cotransporter-2 (SGLT2) inhibitors, and mineralocorticoid receptor (MR) blockers (all binary).

For missing laboratory data, we first attempted to impute values using the nearest available measurement within one month before or after the visit. For remaining missing values, we employed missing indicators in our analysis^10^. The proportions of missing data are provided in Supplement Table 2.

### Model development approaches

We employed three distinct approaches to predict eGFR trajectories up to three years from the index date.: (1) a conventional linear regression model using historical eGFR measurements prior to the index date, which has been traditionally used in clinical settings, (2) a gradient boosting model using Light Gradient Boosting Machine (LightGBM), which is known for its efficiency and high performance in handling structured data^11^, and (3) a recursive prediction model using Long Short-Term Memory (LSTM) networks, which are specifically designed to process sequential data^12^. Further details on model development methodologies are provided in Supplement Note 1.

### Hyperparameter tuning

Next, using only the training data (80%), five-fold cross-validation was performed. Model performance was evaluated in each fold, and the combination of hyperparameters that achieved the best performance was selected. Model hyperparameters were optimized during the training process using the 5-fold cross-validation structure described above. For both LightGBM and LSTM models, we employed Bayesian optimization for hyperparameter tuning. Performance measures, including RMSE and coefficient of determination, were calculated as the average across all validation folds. Bayesian optimization method was used for both models. A comprehensive summary of the necessary hyperparameters for both machine learning algorithms is provided in the Supplement Table 3.

### Evaluation criteria

We evaluated the final model’s predictive accuracy using the following systematic approach. Using the optimal hyperparameters obtained from the tuning process, the final model was constructed. This final model was then applied to the held-out test data (20%), which had never been used in training or tuning, and the reported RMSE value was calculated.The model was designed to predict eGFR values at three annual time points over a three-year period from the initial measurement.

For both predicted and actual eGFR values, we calculated individual patient-specific slopes using linear regression analysis across four time points: baseline and years 1, 2, and 3. The model’s performance was evaluated using the Root Mean Square Error (RMSE) between the predicted and actual slopes, calculated as:

$$\:\mathrm{RMSE}=\sqrt{\frac{1}{n}{\sum\:}_{i=1}^{n}{\left(\mathrm{predicted\:}{\mathrm{slope}}_{i}-\mathrm{actual\:}{\mathrm{slope}}_{i}\right)}^{2}}$$

where n is the number of patients in the test dataset, predicted slope is the slope calculated from the model’s predictions, and actual slope is the slope derived from the observed eGFR values.

## Results

### Baseline data

**Table 1** shows the baseline characteristics of the study population. The median age of participants was 69.0 years [IQR: 62.0–77.0], and 52% (5,493/10,474) of the cohort were male. The Median baseline eGFR was 52.7 mL/min/1.73 m² [IQR: 44.7–57.8]. Laboratory values included serum creatinine of 1.0 mg/dL [IQR: 0.8–1.2], plasma albumin of 4.1 g/dL [IQR: 3.9–4.4], serum sodium of 141.0 mEq/L [IQR: 140.0–143.0.0.0], and serum potassium of 4.4 mEq/L [IQR: 4.1–4.7]. Regarding urinary findings, 52% (2,346/4,471) of patients had positive proteinuria by dipstick test, with median urine protein of 21.0 g/gCr [IQR: 7.0–67.5.0.5]. Comorbidities and medications were also documented: diabetes mellitus was present in 75% of patients and hypertension in 71%. Regarding medications, 24% of patients were prescribed RAS inhibitors, 2.2% were on MR blockers, and 0.4% were on SGLT2 inhibitors at baseline. With a median follow-up period of 6 years, longitudinal eGFR measurements showed a gradual decline over the three-year follow-up period: median eGFR at 1 year was 52.7 mL/min/1.73 m² [IQR: 44.5–59.7], at 2 years was 51.9 mL/min/1.73 m² [IQR: 43.0–59.0], and at 3 years was 51.2 mL/min/1.73 m² [IQR: 42.0–58.5.0.5].

**Table 1:** Patient characteristics. Baseline characteristics. Clinical and demographic characteristics of the study population at baseline, including laboratory values, comorbidities, and medications. eGFR, estimated Glomerular Filtration Rate; CKD, Chronic Kidney Disease; IQR, Interquartile Range; RAS, Renin-Angiotensin System; SGLT2, Sodium-Glucose Cotransporter-2; MR, Mineralocorticoid Receptor

Characteristics	Overall n = 10474**^1^
Age	69.0 [62.0 - 77.0]
Gender	5,493 / 10,474 (52%)
Baseline eGFR (mL/min/1.73m²)	52.7 [44.7 - 57.8]
Serum Creatinine (mg/dL)	1.0 [0.8 - 1.2]
Plasma Albumin (g/dL)	4.1 [3.9 - 4.4]
Serum Sodium (mEq/L)	141.0 [140.0 - 143.0]
Serum Potassium (mEq/L)	4.4 [4.1 - 4.7]
Urine Protein Qualitative	2,346 / 4,471 (52%)
Urine Protein (g/gCr)	21.0 [7.0 - 67.5]
Diabetes Mellitus	7,864 / 10,474 (75%)
Hypertension	7,470 / 10,474 (71%)
SGLT2 inhibitor	46 / 10,474 (0.4%)
RAS inhibitor	2,548 / 10,474 (24%)
MR Blocker	226 / 10,474 (2.2%)
1-year eGFR	52.7 [44.5 - 59.7]
2-year eGFR	51.9 [43.0 - 59.0]
3-year eGFR	51.2 [42.0 - 58.5]
Number of eGFR measurements prior to index	4.00 [2.58 – 5.42]
Observation period prior to index (year)	0.708 [1.07- 0.35]
^1^Median [IQR]; n / N (%)	

### Comparison of model performance

**Fig. 3:**
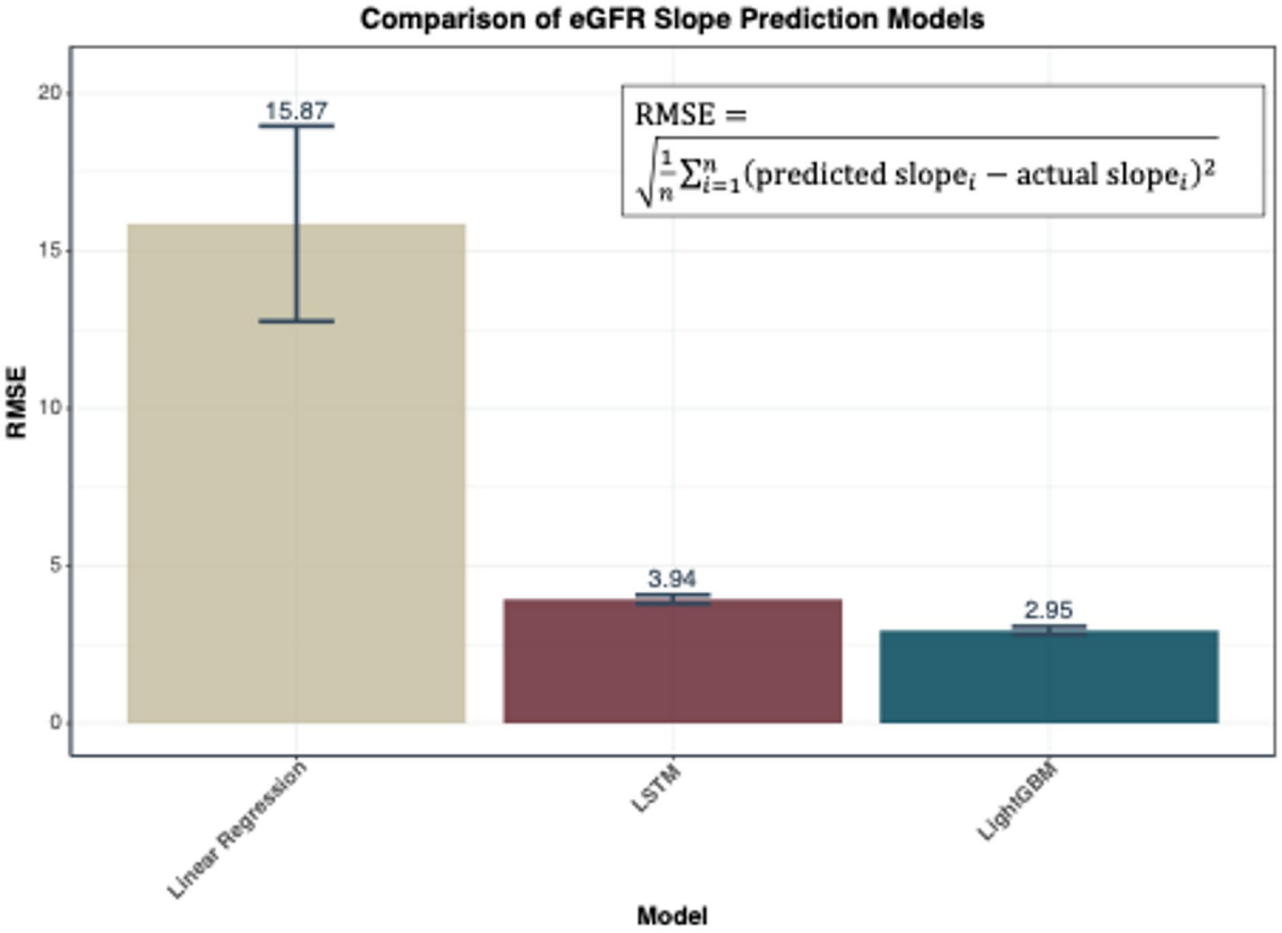
Comparison of eGFR Slope Prediction Models. Performance comparison of three prediction approaches using RMSE. Abbreviations: eGFR, estimated Glomerular Filtration Rate; RMSE, Root Mean Square Error; LSTM, Long Short-Term Memory; LightGBM, Light Gradient Boosting Machine.

We compared three different approaches to predicting three-year eGFR trajectories **(**Fig. 3). The linear regression model used only pre-baseline eGFR measurements for prediction, whereas both the LightGBM and LSTM models used baseline eGFR values together with patient characteristics to predict future trajectories. These predictions were compared with the actual slope calculated using linear regression analysis of quarterly measured eGFR values over three years at baseline, years 1, 2 and 3.

The conventional linear regression model showed an RMSE of 15.87 mL/min/1.73 m²/year. The LSTM model, which included additional clinical parameters, achieved an RMSE of 3.94 mL/min/1.73 m²/year, while the LightGBM model showed the highest accuracy with an RMSE of 2.95 mL/min/1.73 m²/year. To assess the distribution of individual prediction errors, we calculated the absolute difference between predicted and actual slopes for each patient and determined the percentile ranges. For the LightGBM model (RMSE = 2.95 mL/min/1.73 m²/year), 95% of individual predictions had absolute errors within 6.6 mL/min/1.73 m²/year (2.5th to 97.5th percentile range: 0.07 to 6.63 mL/min/1.73 m²/year).

### Implementation of the models in clinical settings

**Fig. 4:**
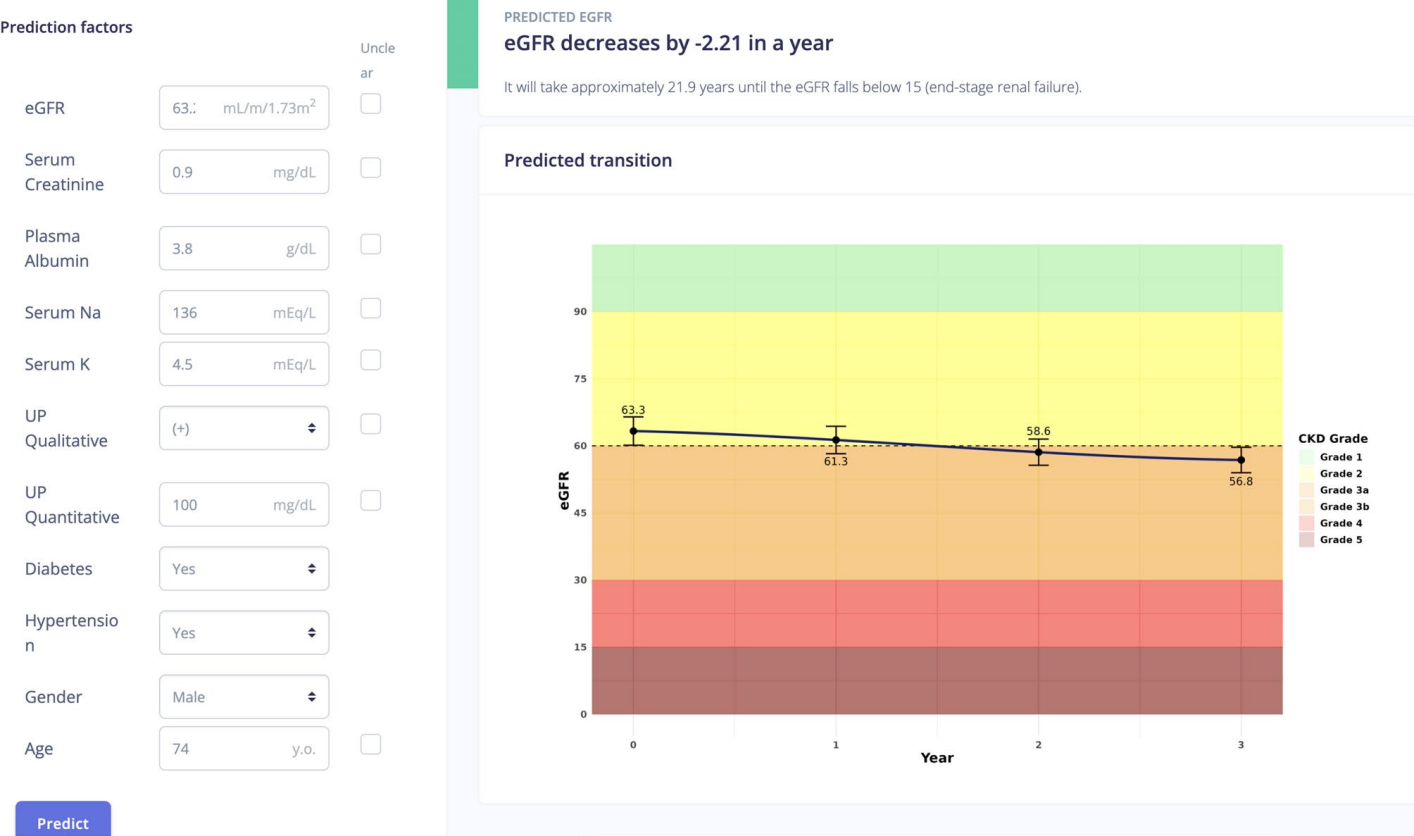
Application of Prediction eGFR Slope. Web-based interface demonstration for clinical implementation of the prediction model. Abbreviations: eGFR, estimated Glomerular Filtration Rate. (URL:https://app.j-ka.or.jp/)

Figure 4 shows the web-based application we developed incorporating the LightGBM model, which demonstrated the highest prediction accuracy among the three approaches. The application (URL: https://app.j-ka.or.jp/) provides an intuitive interface for healthcare providers to input patient data and visualize predicted eGFR trajectories. When clinicians input patient characteristics and laboratory data, the application displays the predicted eGFR slope values (top right panel) and predicted future trajectories over a three-year period (bottom right panel).

## Discussion

**Fig. 5:**
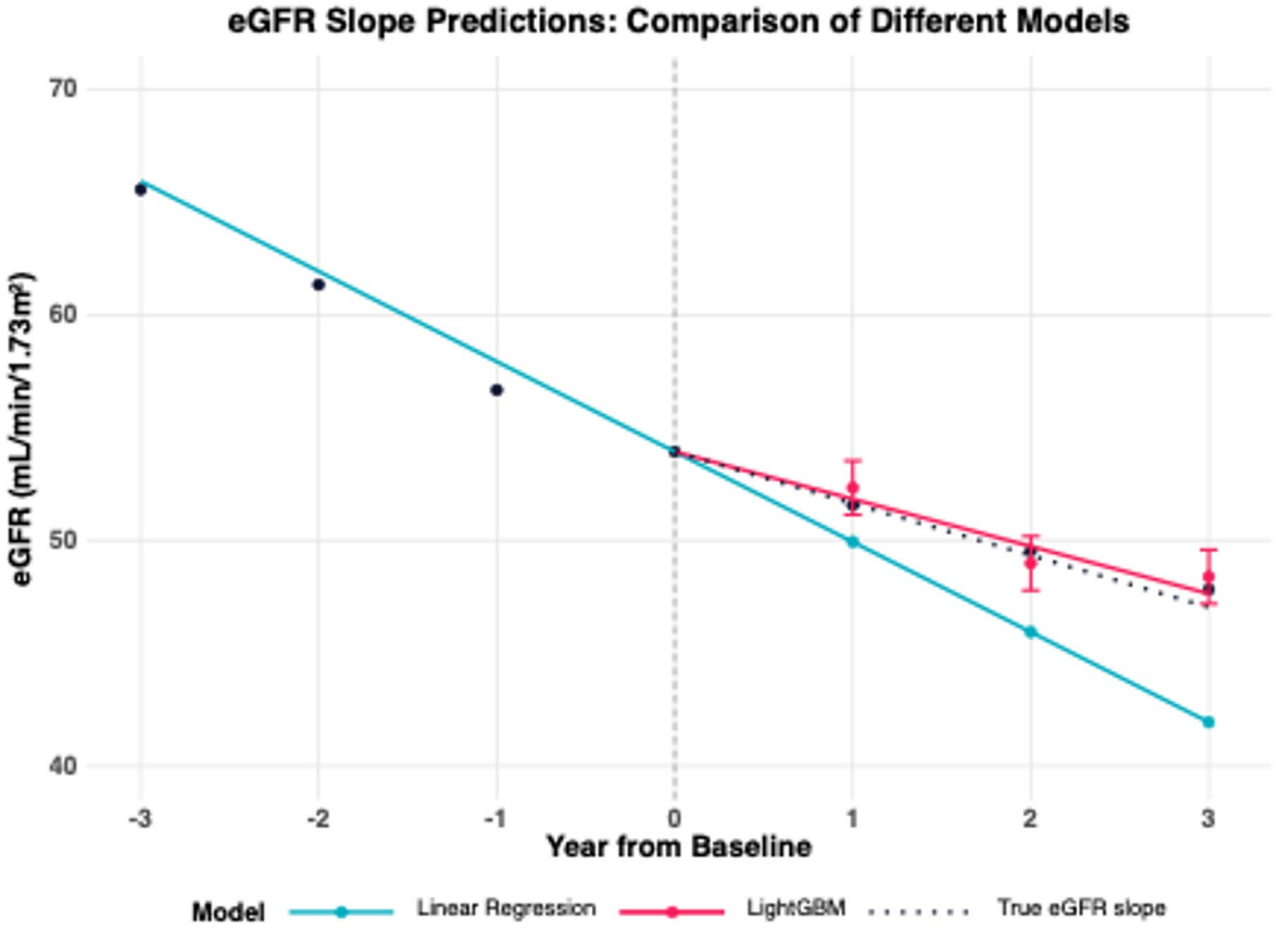
Conceptual diagram of the results of this study. A conceptual diagram of the results of this study. Y-axis: eGFR (estimated Glomerular Filtration Rate) X-axis: Year.,Blue Line: Predictions from Linear Regression., Red Line: Predictions from LightGBM., Dashed Line: True eGFR slope. Abbreviations: eGFR, estimated Glomerular Filtration Rate, LightGBM, Light Gradient Boosting Machine.

A conceptual diagram of the results of this study is shown in Fig. 5. We have successfully developed a prediction model for eGFR slope using a nationwide CKD cohort, achieving a 3-year slope error of 2.8 mL/min/1.73m^2^ in RMSE. This represents a significant improvement over the conventional linear regression model, which showed an error of 17.2 mL/min/1.73m^2^. The model’s ability to generate predictions using single time-point data, commonly available in clinical practice, makes it particularly valuable. When baseline data was included in the multiple linear regression model, the RMSE improved significantly to 3.07 compare to linear regression. This result suggests that baseline data may have a greater influence on future eGFR slope than past eGFR slope. The model was designed primarily with baseline data so that it could be used in PCP practice settings. This limited the input information to base line data, which raised concerns about accuracy, but the RMSE remained satisfactory. The results of this are consistent with the report of Kovesdy et al.^13^. Also shows regression coefficients in the Supplement Table 1. Furthermore, we have developed a web-based application that effectively visualizes predicted results for both PCPs and patients.

To translate the RMSE into its impact on estimated future eGFR values: given that the median baseline eGFR in our cohort was 52.7 mL/min/1.73 m², an RMSE of 2.95 mL/min/1.73 m²/year translates to approximately ± 8.9 mL/min/1.73 m² in the estimated eGFR at year 3 (calculated as 2.95 × 3 years). For the majority of patients (95%), the prediction error in 3-year eGFR would be within approximately ± 20 mL/min/1.73 m² (6.6 × 3 years). This level of accuracy remains clinically meaningful for guiding decisions regarding specialist referral and treatment intensification in primary care settings. In contrast, the conventional linear regression approach (RMSE = 15.87 mL/min/1.73 m²/year) would result in 3-year eGFR prediction errors of approximately ± 48 mL/min/1.73 m², which substantially exceeds clinically acceptable margins for patient management decisions.

Recent randomized controlled trials (RCTs) investigating renoprotective effects have increasingly focused on eGFR slope changes as a surrogate endpoint. Multiple studies have highlighted the importance of both early albuminuria changes and GFR slope as surrogate endpoints for kidney disease progression. Major trials such as CREDENCE and EMPA-KIDNEY have incorporated eGFR slope as an exploratory and tertiary outcome, respectively, while maintaining kidney composite outcomes as their primary endpoint^14,15^. More recently, Phase 3 trials have begun using these surrogate endpoints as primary outcomes^16^. The evidence supporting GFR slopes as surrogate endpoints is more robust compared to albuminuria changes, suggesting that routine eGFR slope assessment may be more clinically valuable than UACR monitoring.

The eGFR slope provides a tangible measure of CKD progression and addresses crucial needs of PCPs, particularly in facilitating collaboration with nephrologists. Recent developments include tools for visualizing long-term eGFR slopes^17^, with studies demonstrating increased specialist referrals following PCPs’ use of such applications. Our prediction model offers enhanced temporal flexibility compared to traditional approaches. While calculating eGFR slope typically requires multiple measurements over time, potentially delaying prognosis assessment^18^, our model can generate predictions at any point using available historical health data. For PCPs managing patients with CKD, the critical information needs include predicting disease progression and assessing the risk of near-term kidney failure. Our prediction model was deliberately designed to rely on cross-sectional data, although it is well recognized that including a wider range of variables could further improve accuracy. Indeed, Iwagami et al. successfully increased the accuracy of their XGBoost model to an RMSE of 0.78^19^. On the other hand, predictive accuracy and clinical usability often involve a trade-off, suggesting that different prediction models may be required to meet varying clinical needs. The visualization of kidney function changes through eGFR slope not only serves PCPs’ needs but also helps raise patient awareness in early CKD stages.

Numerous models exist for predicting kidney failure, with improving accuracy. The Kidney Failure Risk Equations, developed using Cox proportional hazards models for patients with CKD, and the survival index developed by DOPPS for hemodialysis patients represent significant advances^20–23^. The Kidney Failure Risk Equations, derived from 3,449 CKD G3-5 patients in Ontario, Canada^24^, was externally validated using British Columbia data^25^. The Risk Prediction Equations, developed using data from 5,222,711 people across 28 countries in the CKD Prognosis Consortium^26^, predicts 5-year eGFR decline. While machine learning models have been developed to predict eGFR slope stability^27^, these typically provide binary predictions rather than quantitative assessments. In actual clinical practice, most patients do not progress to ESKD within three years. In this context, eGFR slope is a useful metric for assessing long-term kidney prognosis. Therefore, for patients with early-stage CKD commonly managed in primary care, our eGFR slope–based model may provide greater clinical utility. Conversely, for more advanced CKD patients typically seen in nephrology settings, models predicting ESKD may be more appropriate.

Our study has several limitations. SS-MIX2 system database lacks information on CKD etiology, body mass index, and blood pressure levels. During model development, we evaluated the impact of SGLT2 inhibitors and MR blocker on eGFR slope predictions. However, their limited prescription rates in Japan during our study period (2014–2022) resulted in minimal predictive contribution. Given their negligible impact on model performance and to enhance practical usability, we opted to exclude these medication factors from the web-based application for clinical use. Additional limitation is selection bias from requiring baseline and at least three follow-up eGFR measurements, which may exclude patients with poor healthcare access. While this improves eGFR slope estimation, it limits generalizability. However, since interventions target patients engaged in care, this enhances clinical relevance. The model may also overestimate outcomes for those with irregular follow-up. External validation in more diverse datasets is needed to assess broader applicability. Cross-validation is a widely used method to mitigate overfitting; however, it does not ensure external validity. Future work should focus on prospective evaluation through broader implementation of this application and the accumulation of real-world data. The purpose of this study was to develop a model predicting the eGFR slope. Therefore, the training data were restricted to patients with at least three eGFR measurements. In real-world clinical practice, however, many patients do not undergo sufficient eGFR testing. The validity of applying our model to such patients remains uncertain.

The practical implementation of prediction tools through applications is crucial for clinical utility. Visualization enhances usefulness for both PCPs and patients, as demonstrated by Kanda et al.‘s machine-learning-based web system for CKD risk prediction and treatment^28^. While existing tools like the Kidney Failure Risk Equations (available at https://kidneyfailurerisk.com/) offer valuable insights, their 5-year prediction window may be insufficient for clinical practice, particularly for early-stage patients with CKD. Our model’s ability to predict actual eGFR slopes and visualize CKD grade transitions provides a distinct advantage in patient education and management.

## Supplementary Information

Below is the link to the electronic supplementary material.

Supplementary Material 1

## Data Availability

The data cannot be fully shared publicly. The reasons are as follows: Data contain potentially sensitive information; Patients did not provide informed consent regarding release of personal data; the Ethics Board of Kawasaki Medical School imposed data restriction. The data are owned by J-CKD-DB project committee. Interested readers may request the data at J-CKD-DB project committee; URL (Japanese), https://j-ckd-db.jp. And the following Email address of the hospital may be useful for readers: jckdext@med.kawasaki-m.ac.jp.
