# Correction: Prediction of estimated glomerular filtration rate slope and kidney prognosis of patients with chronic kidney disease

**DOI:** 10.1038/s41598-026-52954-1

**Published:** 2026-05-12

**Authors:** Hajime Nagasu, Takaya Nakashima, Katsuhito Ihara, Ryo Fujimori, Tadahiro Goto, Daisuke Nitta, Seiji Kishi, Tamaki Sasaki, Naoki Kashihara

**Affiliations:** 1 Department of Nephrology and Hypertension, Kawasaki Medical School, 577 Matsushima, Kurashiki, Okayama, 701-0192 Japan; 2 Department of Anesthesiology and Intensive Care Medicine, Nagasaki University Graduate School of Biomedical Sciences, Nagasaki, Japan; 3 TXP Medical Co. Ltd, Tokyo, Japan; 4 Medicine Division, Nippon Boehringer Ingelheim Co. Ltd, Tokyo, Japan; 5 Department of Health Data Science, Graduate School of Data Science, Yokohama City University, Yokohama, Japan; 6 Kawasaki Geriatric Medical Center, Kawasaki Medical School, Okayama, Japan

Correction to: *Scientific Reports* 10.1038/s41598-026-38246-8, published online 17 February 2026

The original version of this Article contained errors. The Affiliation 7 was incorrectly listed and consequently has been removed. Daisuke Nitta was incorrectly affiliated with the affiliations 1 and 7. As a result, their correct affiliation is listed below:

4. Medicine Division, Nippon Boehringer Ingelheim Co. Ltd, Tokyo, Japan

Additionally, in the original version of this Article the legends for Figs. 1, 2, 3, 4 and 5 were inadvertently switched.

The legend of Figure 1,

“Comparison of eGFR Slope Prediction Models Performance comparison of three prediction approaches using RMSE. Abbreviations: eGFR, estimated Glomerular Filtration Rate; RMSE, Root Mean Square Error; LSTM, Long Short-Term Memory; LightGBM, Light Gradient Boosting Machine.”

now reads:

“Patients flow of this study. The patient data flow used in this study is show. The study used data from J-CKD-DB-Ex from 2014 to 2022. eGFR, estimated glomerular filtration rate.”

The legend of Figure 2,

“Application of Prediction eGFR Slope Web-based interface demonstration for clinical implementation of the prediction model. Abbreviations: eGFR, estimated Glomerular Filtration Rate.”

now reads:

“Definition of measurement windows and data collection points for eGFR Study timeline depicting the index date and follow-up measurement windows for eGFR data collection. Abbreviations: eGFR, estimated Glomerular Filtration Rate.”

The legend of Figure 3,

“Conceptual diagram of the results of this study. A conceptual diagram of the results of this study. Y-axis: eGFR (estimated Glomerular Filtration Rate) X-axis: Year.,Blue Line: Predictions from Linear Regression., Red Line: Predictions from LightGBM., Dashed Line: True eGFR slope. Abbreviations: eGFR, estimated Glomerular Filtration Rate, LightGBM, Light Gradient Boosting Machine.”

now reads:

“Comparison of eGFR Slope Prediction Models Performance comparison of three prediction approaches using RMSE. Abbreviations: eGFR, estimated Glomerular Filtration Rate; RMSE, Root Mean Square Error; LSTM, Long Short-Term Memory; LightGBM, Light Gradient Boosting Machine.”

The legend of Figure 4,

“Patients flow of this study The patient data flow used in this study is show. The study used data from J-CKD-DB-Ex from 2014 to 2022. eGFR, estimated glomerular filtration rate.”

now reads:

“Application of Prediction eGFR Slope Web-based interface demonstration for clinical implementation of the prediction model. Abbreviations: eGFR, estimated Glomerular Filtration Rate. (URL:https://app.j-ka.or.jp/).”

The legend of Figure 5,

“Definition of measurement windows and data collection points for eGFR Study timeline depicting the index date and follow-up measurement windows for eGFR data collection. Abbreviations: eGFR, estimated Glomerular Filtration Rate.”

now reads:

“Conceptual diagram of the results of this study. A conceptual diagram of the results of this study. Y-axis: eGFR (estimated Glomerular Filtration Rate) X-axis: Year.,Blue Line: Predictions from Linear Regression., Red Line: Predictions from LightGBM., Dashed Line: True eGFR slope. Abbreviations: eGFR, estimated Glomerular Filtration Rate, LightGBM, Light Gradient Boosting Machine.”

Finally, there was an error in the Results section, under the subheading ‘Implementation of the models in clinical settings’,

“The application provides an intuitive interface for healthcare providers to input patient data and visualize predicted eGFR trajectories.”

now reads:

“The application (URL:https://app.j-ka.or.jp/) provides an intuitive interface for healthcare providers to input patient data and visualize predicted eGFR trajectories.”

The original Article has been corrected.

