# Supplementary material for: Prediction of estimated glomerular filtration rate slope and kidney prognosis of patients with chronic kidney disease
Source: Sci Rep. 2026 Feb 17;16:8883. doi: 10.1038/s41598-026-38246-8 (PMC12988208; doi:10.1038/s41598-026-38246-8)
Supplement: Supplementary file 1 — Supplementary Material 1 [file 41598_2026_38246_MOESM1_ESM.docx]

**Supplement Table 1. Regression coefficients of multiple regression model**

| **Variable** | **Coefficient** | **p-value** |
| --- | --- | --- |
| **Historical Parameters** |  |  |
| Historical slope | -0.159*** | <0.001 |
| Historical intercept | 0.964*** | <0.001 |
| **Laboratory Parameters at Baseline** |  |  |
| Age | 0.055** | 0.007 |
| Serum Creatinine | 0.316 | 0.405 |
| Plasma Albumin | 2.559*** | <0.001 |
| Serum Sodium | -0.007 | 0.940 |
| Serum Potassium | -0.863 | 0.142 |
| Qualitative urine protein | -3.338*** | <0.001 |
| Quantitative urine protein | -0.002 | 0.154 |
| **Clinical Diagnoses at Baseline** |  |  |
| Diabetes | 0.959 | 0.701 |
| Hypertension | -0.507 | 0.821 |
| **Demographic Factors** |  |  |
| Gender | -1.747** | 0.002 |
| **Medications at Baseline** |  |  |
| SGLT2 inhibitors | -6.783* | 0.036 |
| RAS inhibitors | -0.868 | 0.127 |
| MR blockers | -0.966 | 0.551 |

- Significance levels: *p<0.05, **p<0.01, ***p<0.001
- Intercept term (coefficient: -7.846, p-value: 0.581) is not shown in categorical sections
- Historical parameters were derived from simple linear regression of eGFR measurements taken before baseline
- Negative coefficients indicate factors associated with greater eGFR decline

**Abbreviations:** eGFR, estimated Glomerular Filtration Rate; SGLT2, Sodium-Glucose Cotransporter-2; RAS, Renin-Angiotensin System; MR, Mineralocorticoid Receptor

**Supplement Table 2: Missing Data**

| **Variable** | **Missing Values (n)** | **Missing Values (%)** |
| --- | --- | --- |
| **Training Dataset (n = 8,378)** | | |
| Quantitative Urine Protein | 6713 | 80.1 |
| Qualitative Urine Protein | 4823 | 57.6 |
| Serum Sodium | 2567 | 30.6 |
| Plasma Albumin | 2440 | 29.1 |
| Serum Potassium | 1979 | 23.6 |
| Serum Creatinine | 1860 | 22.2 |
| Gender | 0 | 0.0 |
| Age | 0 | 0.0 |
| Baseline eGFR | 0 | 0.0 |
| Diabetes | 0 | 0.0 |
| Hypertension | 0 | 0.0 |
| SGLT2 inhibitors | 0 | 0.0 |
| RAS inhibitors | 0 | 0.0 |
| MR blockers | 0 | 0.0 |
| eGFR at 1 year | 0 | 0.0 |
| eGFR at 2 years | 0 | 0.0 |
| eGFR at 3 years | 0 | 0.0 |
| **Test Dataset (n = 2,096)** | | |
| Quantitative Urine Protein | 1635 | 78.0 |
| Qualitative Urine Protein | 1180 | 56.3 |
| Serum Sodium | 620 | 29.6 |
| Plasma Albumin | 598 | 28.5 |
| Serum Potassium | 475 | 22.7 |
| Serum Creatinine | 471 | 22.5 |
| Gender | 0 | 0.0 |
| Age | 0 | 0.0 |
| Baseline eGFR | 0 | 0.0 |
| Diabetes | 0 | 0.0 |
| Hypertension | 0 | 0.0 |
| SGLT2 inhibitors | 0 | 0.0 |
| RAS inhibitors | 0 | 0.0 |
| MR blockers | 0 | 0.0 |
| eGFR at 1 year | 0 | 0.0 |
| eGFR at 2 years | 0 | 0.0 |
| eGFR at 3 years | 0 | 0.0 |

**Abbreviations:** eGFR, estimated Glomerular Filtration Rate; SGLT2, Sodium-Glucose Cotransporter-2; RAS, Renin-Angiotensin System; MR, Mineralocorticoid Receptor

**Supplement Table 3. Description of Model Parameters**

| Parameter | Description |
| --- | --- |
| **LightGBM Parameters** | |
| Number of trees | Number of decision trees (boosting rounds) to be created |
| Minimum observations per leaf | Minimum number of observations required in each leaf node |
| Maximum tree depth | Maximum allowed depth of each decision tree |
| Learning rate | Step size for each iteration's contribution to the final model |
| Loss reduction | Minimum loss reduction required to make a further partition on a leaf node |
| **LSTM Parameters** | |
| Number of layers | Defines the number of layers in the LSTM model |
| Hidden units/layer | Specifies the number of hidden units in each layer of the LSTM model |
| Number of epochs | Determines the number of times the learning algorithm will work through the entire training dataset |
| Dropout rate | Controls the fraction of units that are randomly dropped during training to prevent overfitting |
| Optimization solver | Specifies the optimization algorithm used for training the LSTM model (e.g., Adam, sgdm, rmsprop) |

Abbreviations: LightGBM, Light Gradient Boosting Machine; LSTM, Long Short-Term Memory

**Supplement Note 1: Model Development Approaches for eGFR Trajectory Prediction**

We employed three distinct approaches to predict eGFR trajectories up to three years from baseline: (1) conventional linear regression, (2) Light Gradient Boosting Machine (LightGBM), and (3) Long Short-Term Memory (LSTM) networks.

**Prediction Methodology**

**eGFR Slope Calculation**

For all models, we calculated the predicted eGFR slope using the least squares method applied to four time points: baseline and predicted values at years 1, 2, and 3. This approach ensures consistency in slope calculation across all prediction methods.

**Linear Regression Model**

The conventional linear regression model used only pre-baseline eGFR measurements to extrapolate future trajectory. This approach represents the standard clinical practice of projecting kidney function decline based on historical measurements alone.

**LightGBM Model**

The LightGBM model employed a stepwise prediction approach:

1. First, it predicted eGFR at year 1 using baseline clinical indicators
2. Then, it used the year 1 prediction along with the calculated rate of change to estimate eGFR at year 2
3. Finally, it used years 1 and 2 predictions along with their rates of change to predict eGFR at year 3

This iterative approach allowed the model to incorporate trajectory information at each prediction step, potentially improving accuracy for later time points.

**LSTM Model**

The LSTM neural network model used a simultaneous prediction approach:

1. 14 baseline clinical indicators were used as input features
2. The model architecture included optimized LSTM layers followed by a dense output layer with 3 nodes
3. This architecture enabled simultaneous prediction of eGFR values at years 1, 2, and 3

The LSTM's ability to model complex temporal dependencies allowed it to generate coordinated predictions across all three time points.

**Data Preprocessing and Missing Value Handling**

All models incorporated a consistent approach to handling missing data:

1. We first attempted to impute missing laboratory values using the nearest available measurement within one month
2. For remaining missing values, we employed missing indicators (-999999) in our analysis
3. Numerical features were standardized using means and standard deviations calculated from non-missing values

This preprocessing strategy ensured that models could handle real-world clinical data with inherent incompleteness.
